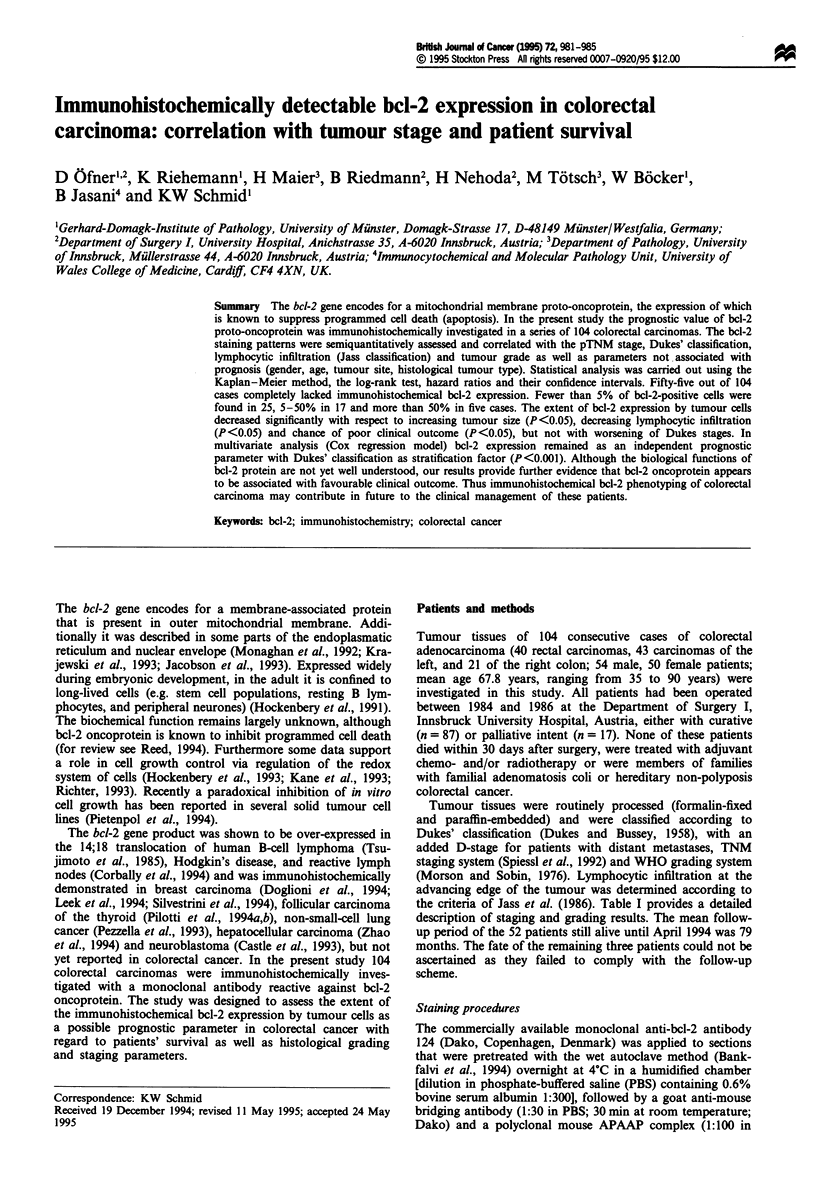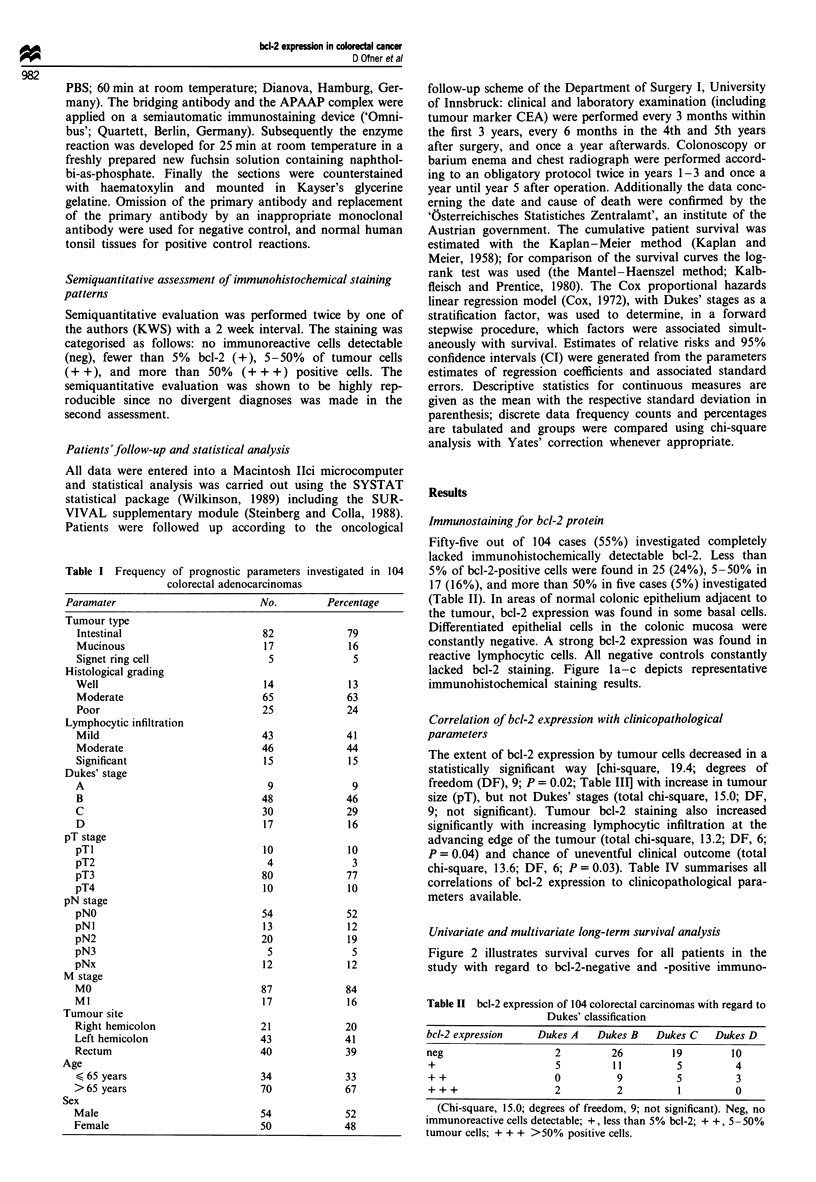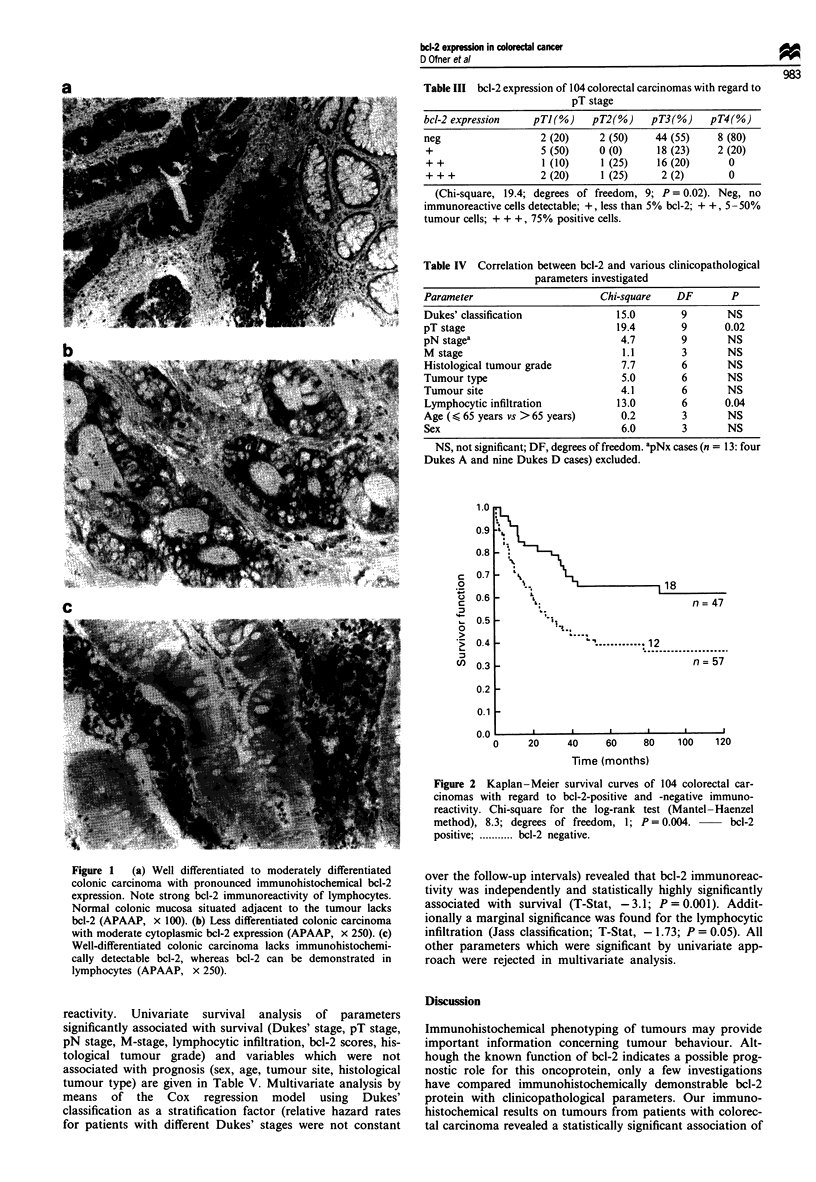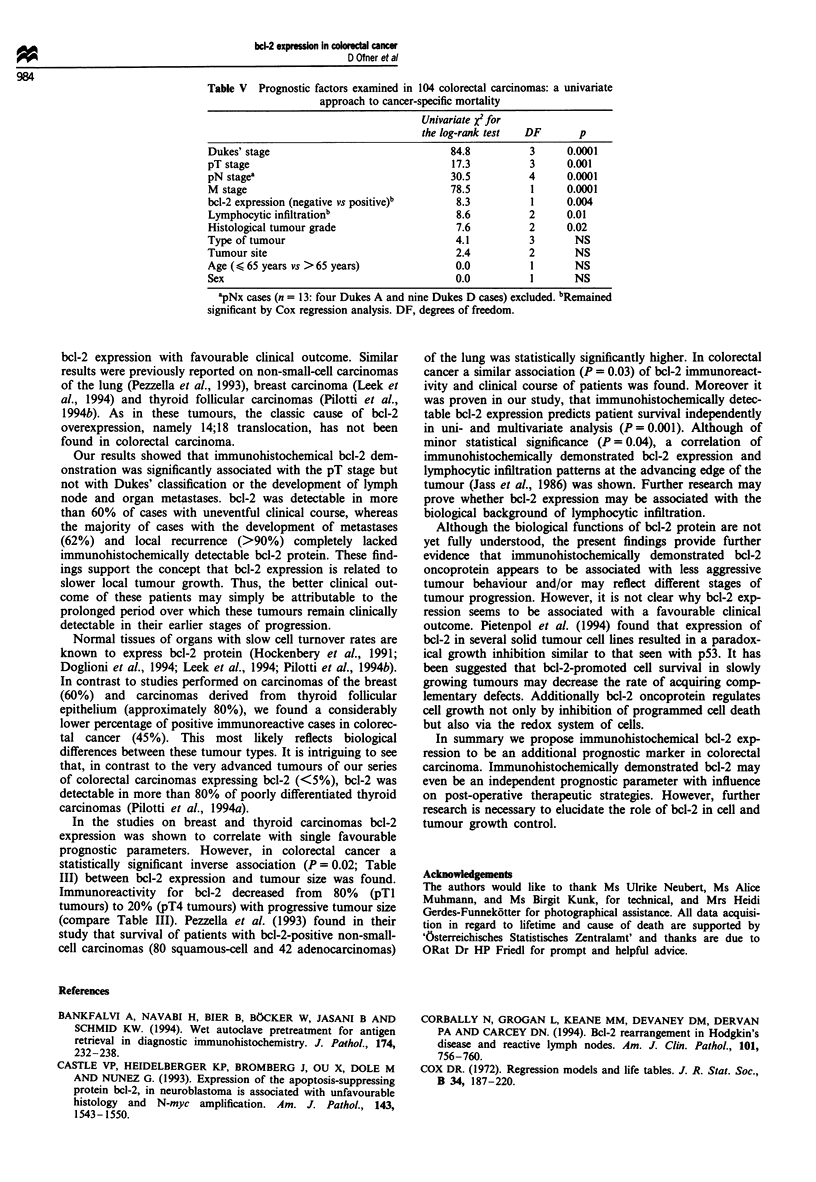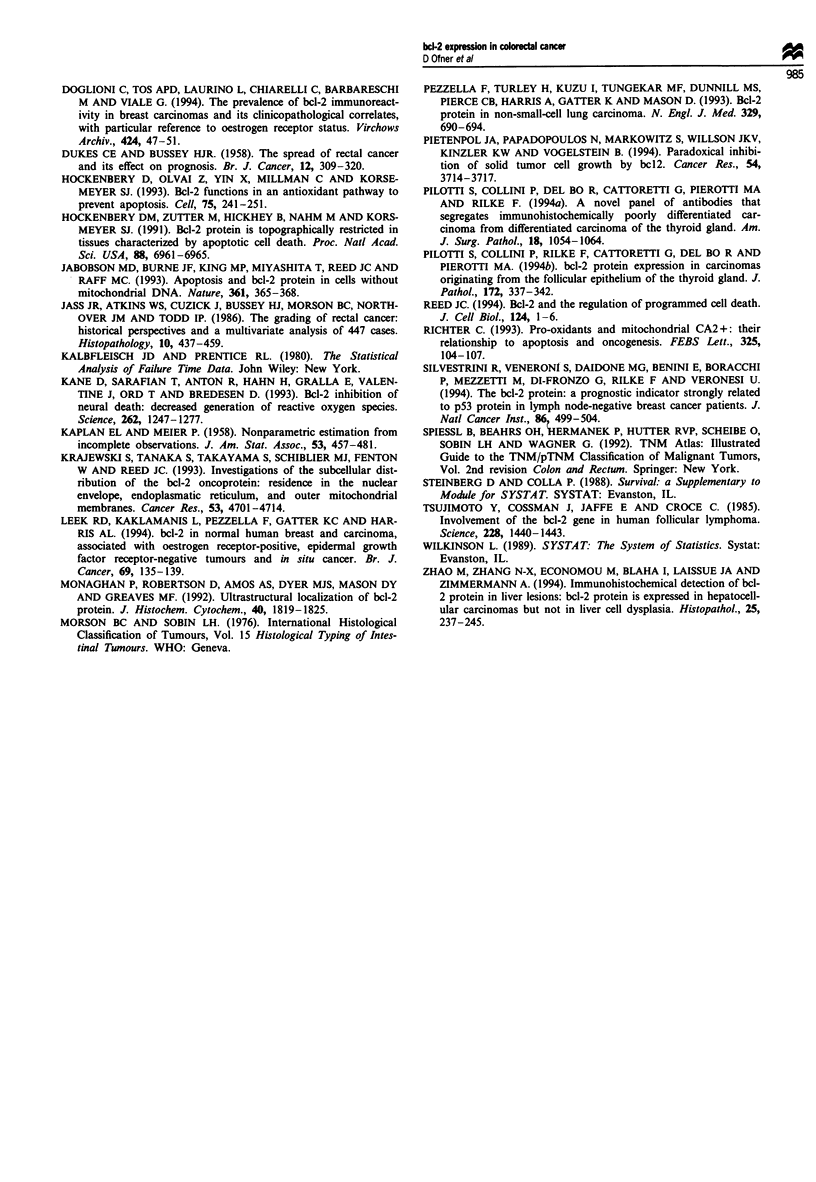# Immunohistochemically detectable bcl-2 expression in colorectal carcinoma: correlation with tumour stage and patient survival.

**DOI:** 10.1038/bjc.1995.446

**Published:** 1995-10

**Authors:** D. Ofner, K. Riehemann, H. Maier, B. Riedmann, H. Nehoda, M. Tötsch, W. Böcker, B. Jasani, K. W. Schmid

**Affiliations:** Gerhard-Domagk-Institute of Pathology, University of Münster, Münster, Westfalia, Germany.

## Abstract

**Images:**


British Journal of Cancer (1995) 72, 981–985

© 1995 Stockton Press All rights reserved 0007–0920/95 $12.00

### Immunohistochemically detectable bcl-2 expression in colorectal carcinoma: correlation with tumour stage and patient survival

D Öfner[1,2], K Riehemann[1], H Maier[3], B Riedmann[2], H Nehoda[2], M Tötsch[3], W Böcker[1], B Jasani[4] and KW Schmid[1]

[1]*Gerhard-Domagk-Institute of Pathology, University of Münster, Domagk-Strasse 17, D-48149 Münster/Westfalia, Germany;* [2]*Department of Surgery I, University Hospital, Anichstrasse 35, A-6020 Innsbruck, Austria;* [3]*Department of Pathology, University of Innsbruck, Müllerstrasse 44, A-6020 Innsbruck, Austria;* [4]*Immunocytochemical and Molecular Pathology Unit, University of Wales College of Medicine, Cardiff, CF4 4XN, UK.*

**Summary** The *bcl-2* gene encodes for a mitochondrial membrane proto-oncoprotein, the expression of which is known to suppress programmed cell death (apoptosis). In the present study the prognostic value of bcl-2 proto-oncoprotein was immunohistochemically investigated in a series of 104 colorectal carcinomas. The bcl-2 staining patterns were semiquantitatively assessed and correlated with the pTNM stage, Dukes' classification, lymphocytic infiltration (Jass classification) and tumour grade as well as parameters not associated with prognosis (gender, age, tumour site, histological tumour type). Statistical analysis was carried out using the Kaplan–Meier method, the log-rank test, hazard ratios and their confidence intervals. Fifty-five out of 104 cases completely lacked immunohistochemical bcl-2 expression. Fewer than 5% of bcl-2-positive cells were found in 25, 5–50% in 17 and more than 50% in five cases. The extent of bcl-2 expression by tumour cells decreased significantly with respect to increasing tumour size ($P<0.05$), decreasing lymphocytic infiltration ($P<0.05$) and chance of poor clinical outcome ($P<0.05$), but not with worsening of Dukes stages. In multivariate analysis (Cox regression model) bcl-2 expression remained as an independent prognostic parameter with Dukes' classification as stratification factor ($P<0.001$). Although the biological functions of bcl-2 protein are not yet well understood, our results provide further evidence that bcl-2 oncoprotein appears to be associated with favourable clinical outcome. Thus immunohistochemical bcl-2 phenotyping of colorectal carcinoma may contribute in future to the clinical management of these patients.

**Keywords:** bcl-2; immunohistochemistry; colorectal cancer

The *bcl-2* gene encodes for a membrane-associated protein that is present in outer mitochondrial membrane. Additionally it was described in some parts of the endoplasmatic reticulum and nuclear envelope (Monaghan *et al.*, 1992; Krajewski *et al.*, 1993; Jacobson *et al.*, 1993). Expressed widely during embryonic development, in the adult it is confined to long-lived cells (e.g. stem cell populations, resting B lymphocytes, and peripheral neurones) (Hockenbery *et al.*, 1991). The biochemical function remains largely unknown, although bcl-2 oncoprotein is known to inhibit programmed cell death (for review see Reed, 1994). Furthermore some data support a role in cell growth control via regulation of the redox system of cells (Hockenbery *et al.*, 1993; Kane *et al.*, 1993; Richter, 1993). Recently a paradoxical inhibition of *in vitro* cell growth has been reported in several solid tumour cell lines (Pietenpol *et al.*, 1994).

The *bcl*-2 gene product was shown to be over-expressed in the 14;18 translocation of human B-cell lymphoma (Tsujimoto *et al.*, 1985), Hodgkin's disease, and reactive lymph nodes (Corbally *et al.*, 1994) and was immunohistochemically demonstrated in breast carcinoma (Doglioni *et al.*, 1994; Leek *et al.*, 1994; Silvestrini *et al.*, 1994), follicular carcinoma of the thyroid (Pilotti *et al.*, 1994*a*,*b*), non-small-cell lung cancer (Pezzella *et al.*, 1993), hepatocellular carcinoma (Zhao *et al.*, 1994) and neuroblastoma (Castle *et al.*, 1993), but not yet reported in colorectal cancer. In the present study 104 colorectal carcinomas were immunohistochemically investigated with a monoclonal antibody reactive against bcl-2 oncoprotein. The study was designed to assess the extent of the immunohistochemical bcl-2 expression by tumour cells as a possible prognostic parameter in colorectal cancer with regard to patients' survival as well as histological grading and staging parameters.

Correspondence: KW Schmid
Received 19 December 1994; revised 11 May 1995; accepted 24 May 1995

### Patients and methods

Tumour tissues of 104 consecutive cases of colorectal adenocarcinoma (40 rectal carcinomas, 43 carcinomas of the left, and 21 of the right colon; 54 male, 50 female patients; mean age 67.8 years, ranging from 35 to 90 years) were investigated in this study. All patients had been operated between 1984 and 1986 at the Department of Surgery I, Innsbruck University Hospital, Austria, either with curative ($n = 87$) or palliative intent ($n = 17$). None of these patients died within 30 days after surgery, were treated with adjuvant chemo- and/or radiotherapy or were members of families with familial adenomatosis coli or hereditary non-polyposis colorectal cancer.

Tumour tissues were routinely processed (formalin-fixed and paraffin-embedded) and were classified according to Dukes' classification (Dukes and Bussey, 1958), with an added D-stage for patients with distant metastases, TNM staging system (Spiessl *et al.*, 1992) and WHO grading system (Morson and Sobin, 1976). Lymphocytic infiltration at the advancing edge of the tumour was determined according to the criteria of Jass *et al.* (1986). Table I provides a detailed description of staging and grading results. The mean follow-up period of the 52 patients still alive until April 1994 was 79 months. The fate of the remaining three patients could not be ascertained as they failed to comply with the follow-up scheme.

### *Staining procedures*

The commercially available monoclonal anti-bcl-2 antibody 124 (Dako, Copenhagen, Denmark) was applied to sections that were pretreated with the wet autoclave method (Bankfalvi *et al.*, 1994) overnight at 4°C in a humidified chamber [dilution in phosphate-buffered saline (PBS) containing 0.6% bovine serum albumin 1:300], followed by a goat anti-mouse bridging antibody (1:30 in PBS; 30 min at room temperature; Dako) and a polyclonal mouse APAAP complex (1:100 in

PBS; 60 min at room temperature; Dianova, Hamburg, Germany). The bridging antibody and the APAAP complex were applied on a semiautomatic immunostaining device ('Omnibus'; Quartett, Berlin, Germany). Subsequently the enzyme reaction was developed for 25 min at room temperature in a freshly prepared new fuchsin solution containing naphthol-bi-as-phosphate. Finally the sections were counterstained with haematoxylin and mounted in Kayser's glycerine gelatine. Omission of the primary antibody and replacement of the primary antibody by an inappropriate monoclonal antibody were used for negative control, and normal human tonsil tissues for positive control reactions.

*Semiquantitative assessment of immunohistochemical staining patterns*

Semiquantitative evaluation was performed twice by one of the authors (KWS) with a 2 week interval. The staining was categorised as follows: no immunoreactive cells detectable (neg), fewer than 5% bcl-2 (+), 5–50% of tumour cells (+ +), and more than 50% (+ + +) positive cells. The semiquantitative evaluation was shown to be highly reproducible since no divergent diagnoses was made in the second assessment.

*Patients' follow-up and statistical analysis*

All data were entered into a Macintosh IIci microcomputer and statistical analysis was carried out using the SYSTAT statistical package (Wilkinson, 1989) including the SURVIVAL supplementary module (Steinberg and Colla, 1988). Patients were followed up according to the oncological follow-up scheme of the Department of Surgery I, University of Innsbruck: clinical and laboratory examination (including tumour marker CEA) were performed every 3 months within the first 3 years, every 6 months in the 4th and 5th years after surgery, and once a year afterwards. Colonoscopy or barium enema and chest radiograph were performed according to an obligatory protocol twice in years 1–3 and once a year until year 5 after operation. Additionally the data concerning the date and cause of death were confirmed by the 'Österreichisches Statistiches Zentralamt', an institute of the Austrian government. The cumulative patient survival was estimated with the Kaplan–Meier method (Kaplan and Meier, 1958); for comparison of the survival curves the log-rank test was used (the Mantel–Haenszel method; Kalbfleisch and Prentice, 1980). The Cox proportional hazards linear regression model (Cox, 1972), with Dukes' stages as a stratification factor, was used to determine, in a forward stepwise procedure, which factors were associated simultaneously with survival. Estimates of relative risks and 95% confidence intervals (CI) were generated from the parameters estimates of regression coefficients and associated standard errors. Descriptive statistics for continuous measures are given as the mean with the respective standard deviation in parenthesis; discrete data frequency counts and percentages are tabulated and groups were compared using chi-square analysis with Yates' correction whenever appropriate.

**Table I** Frequency of prognostic parameters investigated in 104 colorectal adenocarcinomas

| *Paramater* | *No.* | *Percentage* |
|---|---|---|
| Tumour type | | |
| Intestinal | 82 | 79 |
| Mucinous | 17 | 16 |
| Signet ring cell | 5 | 5 |
| Histological grading | | |
| Well | 14 | 13 |
| Moderate | 65 | 63 |
| Poor | 25 | 24 |
| Lymphocytic infiltration | | |
| Mild | 43 | 41 |
| Moderate | 46 | 44 |
| Significant | 15 | 15 |
| Dukes' stage | | |
| A | 9 | 9 |
| B | 48 | 46 |
| C | 30 | 29 |
| D | 17 | 16 |
| pT stage | | |
| pT1 | 10 | 10 |
| pT2 | 4 | 3 |
| pT3 | 80 | 77 |
| pT4 | 10 | 10 |
| pN stage | | |
| pN0 | 54 | 52 |
| pN1 | 13 | 12 |
| pN2 | 20 | 19 |
| pN3 | 5 | 5 |
| pNx | 12 | 12 |
| M stage | | |
| M0 | 87 | 84 |
| M1 | 17 | 16 |
| Tumour site | | |
| Right hemicolon | 21 | 20 |
| Left hemicolon | 43 | 41 |
| Rectum | 40 | 39 |
| Age | | |
| ⩽ 65 years | 34 | 33 |
| > 65 years | 70 | 67 |
| Sex | | |
| Male | 54 | 52 |
| Female | 50 | 48 |

### Results

*Immunostaining for bcl-2 protein*

Fifty-five out of 104 cases (55%) investigated completely lacked immunohistochemically detectable bcl-2. Less than 5% of bcl-2-positive cells were found in 25 (24%), 5–50% in 17 (16%), and more than 50% in five cases (5%) investigated (Table II). In areas of normal colonic epithelium adjacent to the tumour, bcl-2 expression was found in some basal cells. Differentiated epithelial cells in the colonic mucosa were constantly negative. A strong bcl-2 expression was found in reactive lymphocytic cells. All negative controls constantly lacked bcl-2 staining. Figure 1a–c depicts representative immunohistochemical staining results.

*Correlation of bcl-2 expression with clinicopathological parameters*

The extent of bcl-2 expression by tumour cells decreased in a statistically significant way [chi-square, 19.4; degrees of freedom (DF), 9; $P = 0.02$; Table III] with increase in tumour size (pT), but not Dukes' stages (total chi-square, 15.0; DF, 9; not significant). Tumour bcl-2 staining also increased significantly with increasing lymphocytic infiltration at the advancing edge of the tumour (total chi-square, 13.2; DF, 6; $P = 0.04$) and chance of uneventful clinical outcome (total chi-square, 13.6; DF, 6; $P = 0.03$). Table IV summarises all correlations of bcl-2 expression to clinicopathological parameters available.

*Univariate and multivariate long-term survival analysis*

Figure 2 illustrates survival curves for all patients in the study with regard to bcl-2-negative and -positive immuno-

**Table II** bcl-2 expression of 104 colorectal carcinomas with regard to Dukes' classification

| *bcl-2 expression* | *Dukes A* | *Dukes B* | *Dukes C* | *Dukes D* |
|---|---|---|---|---|
| neg | 2 | 26 | 19 | 10 |
| + | 5 | 11 | 5 | 4 |
| + + | 0 | 9 | 5 | 3 |
| + + + | 2 | 2 | 1 | 0 |

(Chi-square, 15.0; degrees of freedom, 9; not significant). Neg, no immunoreactive cells detectable; +, less than 5% bcl-2; + +, 5–50% tumour cells; + + + >50% positive cells.

**Figure 1** (**a**) Well differentiated to moderately differentiated colonic carcinoma with pronounced immunohistochemical bcl-2 expression. Note strong bcl-2 immunoreactivity of lymphocytes. Normal colonic mucosa situated adjacent to the tumour lacks bcl-2 (APAAP, × 100). (**b**) Less differentiated colonic carcinoma with moderate cytoplasmic bcl-2 expression (APAAP, × 250). (**c**) Well-differentiated colonic carcinoma lacks immunohistochemically detectable bcl-2, whereas bcl-2 can be demonstrated in lymphocytes (APAAP, × 250).

reactivity. Univariate survival analysis of parameters significantly associated with survival (Dukes' stage, pT stage, pN stage, M-stage, lymphocytic infiltration, bcl-2 scores, histological tumour grade) and variables which were not associated with prognosis (sex, age, tumour site, histological tumour type) are given in Table V. Multivariate analysis by means of the Cox regression model using Dukes' classification as a stratification factor (relative hazard rates for patients with different Dukes' stages were not constant

**Table III** bcl-2 expression of 104 colorectal carcinomas with regard to pT stage

| *bcl-2 expression* | *pT1(%)* | *pT2(%)* | *pT3(%)* | *pT4(%)* |
|---|---|---|---|---|
| neg | 2 (20) | 2 (50) | 44 (55) | 8 (80) |
| + | 5 (50) | 0 (0) | 18 (23) | 2 (20) |
| + + | 1 (10) | 1 (25) | 16 (20) | 0 |
| + + + | 2 (20) | 1 (25) | 2 (2) | 0 |

(Chi-square, 19.4; degrees of freedom, 9; $P = 0.02$). Neg, no immunoreactive cells detectable; +, less than 5% bcl-2; + +, 5–50% tumour cells; + + +, 75% positive cells.

**Table IV** Correlation between bcl-2 and various clinicopathological parameters investigated

| *Parameter* | *Chi-square* | *DF* | *P* |
|---|---|---|---|
| Dukes' classification | 15.0 | 9 | NS |
| pT stage | 19.4 | 9 | 0.02 |
| pN stage[a] | 4.7 | 9 | NS |
| M stage | 1.1 | 3 | NS |
| Histological tumour grade | 7.7 | 6 | NS |
| Tumour type | 5.0 | 6 | NS |
| Tumour site | 4.1 | 6 | NS |
| Lymphocytic infiltration | 13.0 | 6 | 0.04 |
| Age (⩽ 65 years *vs* > 65 years) | 0.2 | 3 | NS |
| Sex | 6.0 | 3 | NS |

NS, not significant; DF, degrees of freedom. [a]pNx cases ($n$ = 13: four Dukes A and nine Dukes D cases) excluded.

**Figure 2** Kaplan–Meier survival curves of 104 colorectal carcinomas with regard to bcl-2-positive and -negative immunoreactivity. Chi-square for the log-rank test (Mantel–Haenzel method), 8.3; degrees of freedom, 1; $P = 0.004$. —— bcl-2 positive; .......... bcl-2 negative.

over the follow-up intervals) revealed that bcl-2 immunoreactivity was independently and statistically highly significantly associated with survival (T-Stat, −3.1; $P = 0.001$). Additionally a marginal significance was found for the lymphocytic infiltration (Jass classification; T-Stat, −1.73; $P = 0.05$). All other parameters which were significant by univariate approach were rejected in multivariate analysis.

### Discussion

Immunohistochemical phenotyping of tumours may provide important information concerning tumour behaviour. Although the known function of bcl-2 indicates a possible prognostic role for this oncoprotein, only a few investigations have compared immunohistochemically demonstrable bcl-2 protein with clinicopathological parameters. Our immunohistochemical results on tumours from patients with colorectal carcinoma revealed a statistically significant association of

Table V Prognostic factors examined in 104 colorectal carcinomas: a univariate approach to cancer-specific mortality

| | Univariate $\chi^2$ for the log-rank test | DF | p |
|---|---|---|---|
| Dukes' stage | 84.8 | 3 | 0.0001 |
| pT stage | 17.3 | 3 | 0.001 |
| pN stage[a] | 30.5 | 4 | 0.0001 |
| M stage | 78.5 | 1 | 0.0001 |
| bcl-2 expression (negative *vs* positive)[b] | 8.3 | 1 | 0.004 |
| Lymphocytic infiltration[b] | 8.6 | 2 | 0.01 |
| Histological tumour grade | 7.6 | 2 | 0.02 |
| Type of tumour | 4.1 | 3 | NS |
| Tumour site | 2.4 | 2 | NS |
| Age ($\leqslant$ 65 years *vs* $>$65 years) | 0.0 | 1 | NS |
| Sex | 0.0 | 1 | NS |

[a]pNx cases ($n$ = 13: four Dukes A and nine Dukes D cases) excluded. [b]Remained significant by Cox regression analysis. DF, degrees of freedom.

bcl-2 expression with favourable clinical outcome. Similar results were previously reported on non-small-cell carcinomas of the lung (Pezzella *et al.*, 1993), breast carcinoma (Leek *et al.*, 1994) and thyroid follicular carcinomas (Pilotti *et al.*, 1994*b*). As in these tumours, the classic cause of bcl-2 overexpression, namely 14;18 translocation, has not been found in colorectal carcinoma.

Our results showed that immunohistochemical bcl-2 demonstration was significantly associated with the pT stage but not with Dukes' classification or the development of lymph node and organ metastases. bcl-2 was detectable in more than 60% of cases with uneventful clinical course, whereas the majority of cases with the development of metastases (62%) and local recurrence (>90%) completely lacked immunohistochemically detectable bcl-2 protein. These findings support the concept that bcl-2 expression is related to slower local tumour growth. Thus, the better clinical outcome of these patients may simply be attributable to the prolonged period over which these tumours remain clinically detectable in their earlier stages of progression.

Normal tissues of organs with slow cell turnover rates are known to express bcl-2 protein (Hockenbery *et al.*, 1991; Doglioni *et al.*, 1994; Leek *et al.*, 1994; Pilotti *et al.*, 1994*b*). In contrast to studies performed on carcinomas of the breast (60%) and carcinomas derived from thyroid follicular epithelium (approximately 80%), we found a considerably lower percentage of positive immunoreactive cases in colorectal cancer (45%). This most likely reflects biological differences between these tumour types. It is intriguing to see that, in contrast to the very advanced tumours of our series of colorectal carcinomas expressing bcl-2 (<5%), bcl-2 was detectable in more than 80% of poorly differentiated thyroid carcinomas (Pilotti *et al.*, 1994*a*).

In the studies on breast and thyroid carcinomas bcl-2 expression was shown to correlate with single favourable prognostic parameters. However, in colorectal cancer a statistically significant inverse association ($P$ = 0.02; Table III) between bcl-2 expression and tumour size was found. Immunoreactivity for bcl-2 decreased from 80% (pT1 tumours) to 20% (pT4 tumours) with progressive tumour size (compare Table III). Pezzella *et al.* (1993) found in their study that survival of patients with bcl-2-positive non-small-cell carcinomas (80 squamous-cell and 42 adenocarcinomas) of the lung was statistically significantly higher. In colorectal cancer a similar association ($P$ = 0.03) of bcl-2 immunoreactivity and clinical course of patients was found. Moreover it was proven in our study, that immunohistochemically detectable bcl-2 expression predicts patient survival independently in uni- and multivariate analysis ($P$ = 0.001). Although of minor statistical significance ($P$ = 0.04), a correlation of immunohistochemically demonstrated bcl-2 expression and lymphocytic infiltration patterns at the advancing edge of the tumour (Jass *et al.*, 1986) was shown. Further research may prove whether bcl-2 expression may be associated with the biological background of lymphocytic infiltration.

Although the biological functions of bcl-2 protein are not yet fully understood, the present findings provide further evidence that immunohistochemically demonstrated bcl-2 oncoprotein appears to be associated with less aggressive tumour behaviour and/or may reflect different stages of tumour progression. However, it is not clear why bcl-2 expression seems to be associated with a favourable clinical outcome. Pietenpol *et al.* (1994) found that expression of bcl-2 in several solid tumour cell lines resulted in a paradoxical growth inhibition similar to that seen with p53. It has been suggested that bcl-2-promoted cell survival in slowly growing tumours may decrease the rate of acquiring complementary defects. Additionally bcl-2 oncoprotein regulates cell growth not only by inhibition of programmed cell death but also via the redox system of cells.

In summary we propose immunohistochemical bcl-2 expression to be an additional prognostic marker in colorectal carcinoma. Immunohistochemically demonstrated bcl-2 may even be an independent prognostic parameter with influence on post-operative therapeutic strategies. However, further research is necessary to elucidate the role of bcl-2 in cell and tumour growth control.

**Acknowledgements**
The authors would like to thank Ms Ulrike Neubert, Ms Alice Muhmann, and Ms Birgit Kunk, for technical, and Mrs Heidi Gerdes-Funnekötter for photographical assistance. All data acquisition in regard to lifetime and cause of death are supported by 'Österreichisches Statistisches Zentralamt' and thanks are due to ORat Dr HP Friedl for prompt and helpful advice.

### References

BANKFALVI A, NAVABI H, BIER B, BÖCKER W, JASANI B AND SCHMID KW. (1994). Wet autoclave pretreatment for antigen retrieval in diagnostic immunohistochemistry. *J. Pathol.*, **174**, 232–238.

CASTLE VP, HEIDELBERGER KP, BROMBERG J, OU X, DOLE M AND NUNEZ G. (1993). Expression of the apoptosis-suppressing protein bcl-2, in neuroblastoma is associated with unfavourable histology and N-*myc* amplification. *Am. J. Pathol.*, **143**, 1543–1550.

CORBALLY N, GROGAN L, KEANE MM, DEVANEY DM, DERVAN PA AND CARCEY DN. (1994). Bcl-2 rearrangement in Hodgkin's disease and reactive lymph nodes. *Am. J. Clin. Pathol.*, **101**, 756–760.

COX DR. (1972). Regression models and life tables. *J. R. Stat. Soc.*, **B 34**, 187–220.

DOGLIONI C, TOS APD, LAURINO L, CHIARELLI C, BARBARESCHI M AND VIALE G. (1994). The prevalence of bcl-2 immunoreactivity in breast carcinomas and its clinicopathological correlates, with particular reference to oestrogen receptor status. *Virchows Archiv.*, **424,** 47-51.

DUKES CE AND BUSSEY HJR. (1958). The spread of rectal cancer and its effect on prognosis. *Br. J. Cancer*, **12,** 309-320.

HOCKENBERY D, OLVAI Z, YIN X, MILLMAN C AND KORSEMEYER SJ. (1993). Bcl-2 functions in an antioxidant pathway to prevent apoptosis. *Cell*, **75,** 241-251.

HOCKENBERY DM, ZUTTER M, HICKHEY B, NAHM M AND KORSMEYER SJ. (1991). Bcl-2 protein is topographically restricted in tissues characterized by apoptotic cell death. *Proc. Natl Acad. Sci. USA*, **88,** 6961-6965.

JABOBSON MD, BURNE JF, KING MP, MIYASHITA T, REED JC AND RAFF MC. (1993). Apoptosis and bcl-2 protein in cells without mitochondrial DNA. *Nature*, **361,** 365-368.

JASS JR, ATKINS WS, CUZICK J, BUSSEY HJ, MORSON BC, NORTHOVER JM AND TODD IP. (1986). The grading of rectal cancer: historical perspectives and a multivariate analysis of 447 cases. *Histopathology*, **10,** 437-459.

KALBFLEISCH JD AND PRENTICE RL. (1980). *The Statistical Analysis of Failure Time Data.* John Wiley: New York.

KANE D, SARAFIAN T, ANTON R, HAHN H, GRALLA E, VALENTINE J, ORD T AND BREDESEN D. (1993). Bcl-2 inhibition of neural death: decreased generation of reactive oxygen species. *Science*, **262,** 1247-1277.

KAPLAN EL AND MEIER P. (1958). Nonparametric estimation from incomplete observations. *J. Am. Stat. Assoc.*, **53,** 457-481.

KRAJEWSKI S, TANAKA S, TAKAYAMA S, SCHIBLIER MJ, FENTON W AND REED JC. (1993). Investigations of the subcellular distribution of the bcl-2 oncoprotein: residence in the nuclear envelope, endoplasmatic reticulum, and outer mitochondrial membranes. *Cancer Res.*, **53,** 4701-4714.

LEEK RD, KAKLAMANIS L, PEZZELLA F, GATTER KC AND HARRIS AL. (1994). bcl-2 in normal human breast and carcinoma, associated with oestrogen receptor-positive, epidermal growth factor receptor-negative tumours and *in situ* cancer. *Br. J. Cancer*, **69,** 135-139.

MONAGHAN P, ROBERTSON D, AMOS AS, DYER MJS, MASON DY AND GREAVES MF. (1992). Ultrastructural localization of bcl-2 protein. *J. Histochem. Cytochem.*, **40,** 1819-1825.

MORSON BC AND SOBIN LH. (1976). International Histological Classification of Tumours, Vol. 15 *Histological Typing of Intestinal Tumours.* WHO: Geneva.

PEZZELLA F, TURLEY H, KUZU I, TUNGEKAR MF, DUNNILL MS, PIERCE CB, HARRIS A, GATTER K AND MASON D. (1993). Bcl-2 protein in non-small-cell lung carcinoma. *N. Engl. J. Med.* **329,** 690-694.

PIETENPOL JA, PAPADOPOULOS N, MARKOWITZ S, WILLSON JKV, KINZLER KW AND VOGELSTEIN B. (1994). Paradoxical inhibition of solid tumor cell growth by bcl2. *Cancer Res.*, **54,** 3714-3717.

PILOTTI S, COLLINI P, DEL BO R, CATTORETTI G, PIEROTTI MA AND RILKE F. (1994*a*). A novel panel of antibodies that segregates immunohistochemically poorly differentiated carcinoma from differentiated carcinoma of the thyroid gland. *Am. J. Surg. Pathol.*, **18,** 1054-1064.

PILOTTI S, COLLINI P, RILKE F, CATTORETTI G, DEL BO R AND PIEROTTI MA. (1994*b*). bcl-2 protein expression in carcinomas originating from the follicular epithelium of the thyroid gland. *J. Pathol.*, **172,** 337-342.

REED JC. (1994). Bcl-2 and the regulation of programmed cell death. *J. Cell Biol.*, **124,** 1-6.

RICHTER C. (1993). Pro-oxidants and mitochondrial CA2+: their relationship to apoptosis and oncogenesis. *FEBS Lett.*, **325,** 104-107.

SILVESTRINI R, VENERONÍ S, DAIDONE MG, BENINI E, BORACCHI P, MEZZETTI M, DI-FRONZO G, RILKE F AND VERONESI U. (1994). The bcl-2 protein: a prognostic indicator strongly related to p53 protein in lymph node-negative breast cancer patients. *J. Natl Cancer Inst.*, **86,** 499-504.

SPIESSL B, BEAHRS OH, HERMANEK P, HUTTER RVP, SCHEIBE O, SOBIN LH AND WAGNER G. (1992). TNM Atlas: Illustrated Guide to the TNM/pTNM Classification of Malignant Tumors, Vol. 2nd revision *Colon and Rectum.* Springer: New York.

STEINBERG D AND COLLA P. (1988). *Survival: a Supplementary to Module for SYSTAT.* SYSTAT: Evanston, IL.

TSUJIMOTO Y, COSSMAN J, JAFFE E AND CROCE C. (1985). Involvement of the bcl-2 gene in human follicular lymphoma. *Science*, **228,** 1440-1443.

WILKINSON L. (1989). *SYSTAT: The System of Statistics.* Systat: Evanston, IL.

ZHAO M, ZHANG N-X, ECONOMOU M, BLAHA I, LAISSUE JA AND ZIMMERMANN A. (1994). Immunohistochemical detection of bcl-2 protein in liver lesions: bcl-2 protein is expressed in hepatocellular carcinomas but not in liver cell dysplasia. *Histopathol.*, **25,** 237-245.